# Disruption of the FasL/Fas axis protects against inflammation-derived tumorigenesis in chronic liver disease

**DOI:** 10.1038/s41419-019-1391-x

**Published:** 2019-02-08

**Authors:** Francisco Javier Cubero, Marius Maximilian Woitok, Miguel E. Zoubek, Alain de Bruin, Maximilian Hatting, Christian Trautwein

**Affiliations:** 1 Department of Internal Medicine III, University Hospital, RWTH Aachen, Aachen, Germany; 2 Department of Immunology, Ophthalmology and ORL, Complutense University School of Medicine, Madrid, Spain; 3 12 de Octubre Health Research Institute (imas12), Madrid, Spain; 4 Department of Toxicology, Faculty of Health Medicine and Life Sciences, School of Nutrition, Toxicology and Metabolism (NUTRIM), Maastricht University, Maastricht, The Netherlands; 5 Institute of Pathology, Utrecht University, Utrecht, The Netherlands

## Abstract

Fas Ligand (FasL) and Fas (APO-1/CD95) are members of the TNFR superfamily and may trigger apoptosis. Here, we aimed to elucidate the functional role of Fas signaling in an experimental model of chronic liver disease, the hepatocyte-specific NEMO knockout (NEMO^Δhepa^) mice. We generated NEMO^Δhepa^ /Fas^*lpr*^ mice, while NEMO^Δhepa^, NEMO^f/f^ as well as Fas^*lpr*^*animals* were used as controls, and characterized their phenotype during liver disease progression. Liver damage was evaluated by serum transaminases, histological, immunofluorescence procedures, and biochemical and molecular biology techniques. Proteins were detected by western Blot, expression of mRNA by RT-PCR, and infiltration of inflammatory cells was determined by FACs analysis, respectively. Fas^*lpr*^ mutation in NEMO^Δhepa^ mice resulted in overall decreased liver injury, enhanced hepatocyte survival, and reduced proliferation at 8 weeks of age compared with NEMO^Δhepa^ mice. Moreover, NEMO^Δhepa^/Fas^*lpr*^ animals elicited significantly decreased parameters of liver fibrosis, such as Collagen IA1, MMP2, and TIMP1, and reduced proinflammatory macrophages and cytokine expression. At 52 weeks of age, NEMO^Δhepa^/Fas^*lpr*^ exhibited less malignant growth as evidenced by reduced HCC burden associated with a significantly decreased number of nodules and LW/BW ratio and decreased myeloid populations. Deletion of TNFR1 further reduced tumor load of 52-weeks-old NEMO^Δhepa^/Fas^*lpr*^ mice. The functionality of FasL/Fas might affect inflammation-driven tumorigenesis in an experimental model of chronic liver disease. These results help to develop alternative therapeutic approaches and extend the limitations of tumor therapy against HCC.

## Introduction

The transmembrane proteins Fas-Ligand (FasL) and Fas (Fas/ CD95/APO-1) are members of the tumor necrosis factor (TNF) and TNF receptor gene superfamilies (TNFR gene superfamily), respectively, and are expressed in numerous cell types. FasL–Fas interaction plays a crucial role in immune regulation via the ability of FasL to transmit an apoptotic signal to Fas-expressing cells^1^. Particularly, the importance of the FasL/Fas axis in pathophysiology and homeostasis has been well-documented in the liver where these proteins are expressed in hepatocytes, cholangiocytes, activated stellate cells (HSC), and Kupffer cells (KC)^2^.

Downregulation or loss of Fas expression and function is frequently found in the progression of a number of human malignancies, including colon, breast, lung, and liver carcinoma^3,4^. Thus, the FasL–Fas pathway plays a crucial role in tumor initiation and progression. It might be a plausible therapeutic target not only for progression of liver disease, but also hepatocellular carcinoma (HCC).

HCC is the fifth most common solid cancer affecting one million people per year representing the third cause of mortality by cancer worldwide^5,6^. Escape from the immune surveillance may play an important role in liver tumorigenesis. Alteration of the FasL/Fas system is regarded as one of the mechanisms preventing the immune system from rejecting tumor cells^7^. However, little attention has been paid to the role of the Fas/FasL interaction in vivo.

Hepatocyte-specific NEMO knockout (NEMO^Δhepa^) mice are susceptible to spontaneous apoptosis, which leads to chronic hepatocyte injury and regenerative proliferation, constituting a risk factor for cancer development^8^. NEMO^Δhepa^ livers are hypersensitive towards TRAIL stimulation^9^. Moreover, we have also shown that the death receptor TNFR1, but not TRAIL, is involved in determining progression of liver injury in NEMO^Δhepa^^10^.

In the present study, we examined the functional role of deficient FasL/Fas signaling on disease progression and end-stage tumorigenesis in the NEMO^Δhepa^ model.

## Materials and methods

### Housing and Generation of Knockout mice

Animals were maintained in the animal facility of the University Hospital RWTH Aachen according to the German legal requirements. Hepatocyte-specific IKKγ/NEMO mice were generated by crossing loxP site-flanked (floxed [f]) NEMO gene (NEMO^f/f^) with Alfp-cre transgenic animals as described before^9^. These mice were further crossed either with Fas^*lpr*^ knockout mice (purchased from The Jackson Laboratory, Bar Harbor, Maine, USA) to yield NEMO^Δhepa^/Fas^*lpr*^. Finally, we crossed NEMO^Δhepa^/Fas^*lpr*^ with TNFR1^−/−^ mice to further generate NEMO^Δhepa^/Fas^*lpr*^/TNFR1^−/−^ and investigated the impact of TNFR1 in NEMO^Δhepa^/Fas^*lpr*^ mice. To use the proper controls, NEMO^Δhepa^ mice were backcrossed from NEMO^Δhepa^/Fas^*lpr*^, and Fas^*lpr*^ and Fas^*lpr*^/TNFR1^−/−^ were used as controls. Genotypes were confirmed via PCR specific for the respective alleles using DNA from tail biopsies. Progression of liver disease was investigated in male mice between 8–9 weeks and 52–54 weeks of age. Liver injury experiments were performed on mice between 8–9 weeks of age. Serum AST and ALT were measured by standard procedures in the Institute of Clinical Chemistry, University Hospital, RWTH Aachen.

### TUNEL assay

TUNEL test was performed by standard procedures.

### Quantitative real-time PCR

Total RNA was purified from liver tissue using Trizol reagent (Invitrogen, Karlsruhe, Germany). Total RNA (1 μg) was used to synthesize cDNA using SuperScript first-Stand Synthesis System (Invitrogen) and was resuspended in 100 μl of H_2_O. Quantitative real-time PCR was performed using SYBR Green Reagent (Invitrogen) in 7300 real-time PCR system (Applied Biosystem, Darmstadt, Germany). GAPDH expression was used to normalize gene expression, which is represented as times versus WT basal expression. Primer sequences can be provided upon request.

### Histological, immunofluorescence, and immunohistochemical analysis

Livers from mice were harvested and after fixation with 4% PFA, were embedded in paraffin for further histological evaluation. H&E and Sirius Red staining were performed on liver sections. For immunofluorescence analysis, liver cryosections of 5 μm were stained with Ki-67, CD11b (BD Biosciences, Heidelberg, Germany), and F4/80 (BioRad, Hercules, USA). Slides were fixed in 4% PFA at room temperature. Secondary antibody conjugated with Cy3 (Jackson Immunoresearch, West Grove, PA) was used to obtain red fluorescence signal. Mounting solution containing DAPI (Vector Laboratories, Burlingame, CA) was used to counterstain the nuclei of hepatocytes.

### Flow cytometry analysis

Hepatocytes were stained with Annexin V-FITC (BD Biosciences). Immune cells from whole liver were isolated and stained with fluorochrome-conjugated antibodies (CD4-PE, CD8-FITC, CD45-APC-Cy7, CD11b-PE, Ly6G-FITC, and F4/80 Biotin) (BD Biosciences, Heidelberg, Germany). All samples were acquired by flow cytometry (FACS Canto II; BD Biosciences) and analyzed using the Flowjo software.

### Immunoblot analysis

Isolated protein samples were probed with antibodies against RIPK1 (#5389) (Pro-Sci, Poway, CA, USA), Cleaved Caspase-3 (Asp175) (Cell Signaling Technology, Massachusetts, USA), PCNA (clone PC10) (Dianova GmbH, Hamburg, Germany), and GAPDH (MCA4739; 1:5000; AbD SeroTec, Düsseldorf, Germany). As secondary antibodies, anti-rabbit-HRP (#7074; Cell Signaling) and anti-mouse-HRP (#sc-2005; Santa Cruz) were used.

### Statistical analysis

Data are expressed as the mean ± standard error of the mean (SEM). Statistical significance was determined using one-way analysis of variance (ANOVA) with Bonferroni post-hoc test.

## Results

### Generation and characterization of the NEMO^Δhepa^/Fas^*lpr*^ mice

To address the functional relevance of FasL/Fas signaling for chronic disease progression in the NEMO^Δhepa^ model^10,11^, we used mutant Fas mice, the lymphoproliferative (*lpr*) mice (Fas^*lpr*^)^12^. Upon generation (Supplementary Figure 1a), we observed that these animals develop lymphadenopathy by accumulating abnormal T cells and suffer from systemic lupus erythematosus-like autoimmune disease^12^. As expected, Fas^*lpr*^ and NEMO^Δhepa^/Fas^*lpr*^ displayed splenomegalia and presence of lymph nodes in the peritoneum (Supplementary Figure 1b–d).

**Fig. 1:**
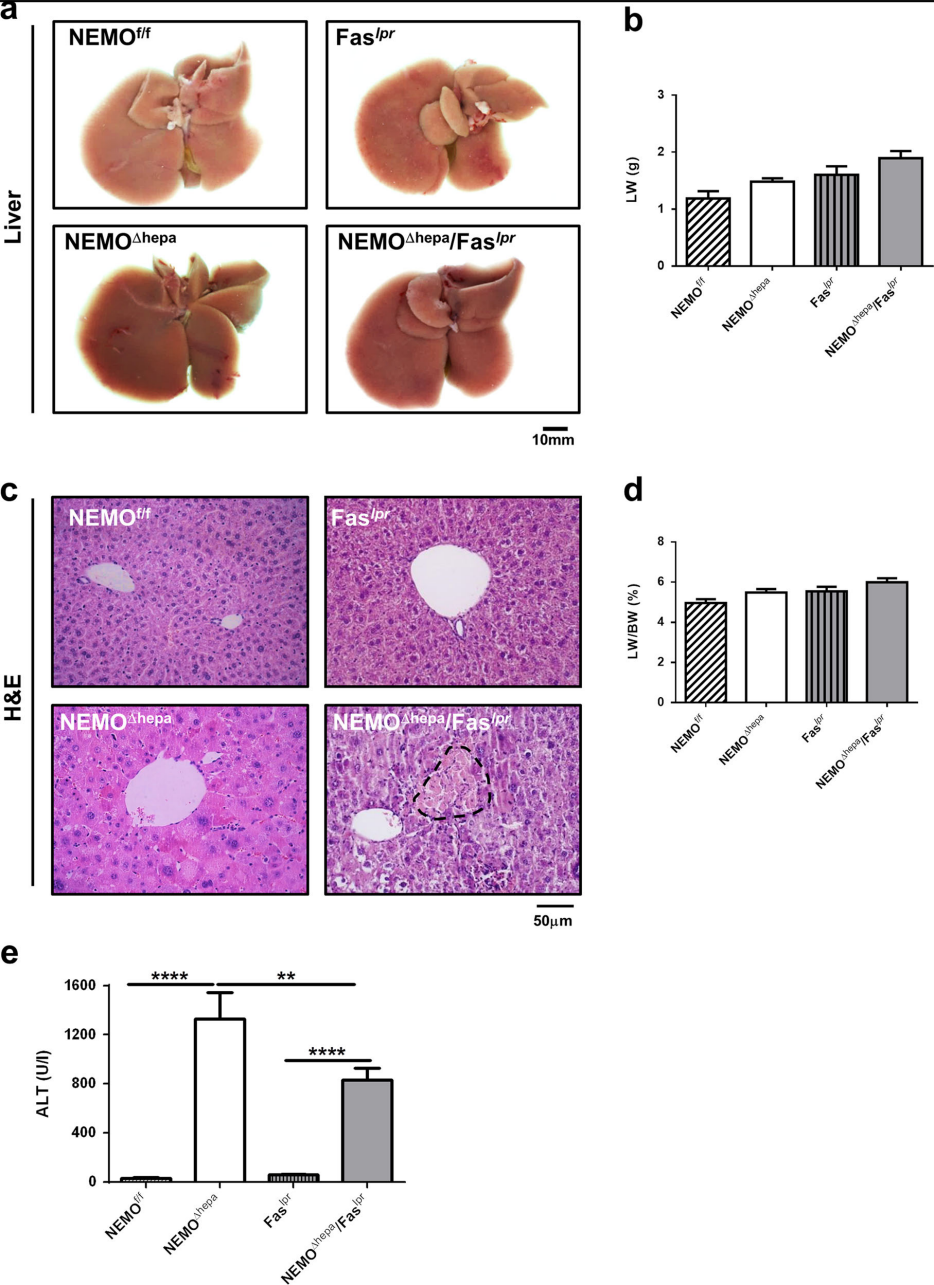
Generation and characterization of 8-week-old NEMO^Δhepa^/Fas^*lpr*^ mice. **a** Macroscopic appearance of livers 8-week-old NEMO^f/f^, NEMO^Δhepa^, Fas^*lpr*^, and NEMO^Δhepa^/Fas^*lpr*^. The liver (LW) (**b**) was calculated and represented. **c** Representative H&E staining of liver sections of 8-week-old animals. **d** The liver versus the body weight (LW/BW) ratio was calculated and represented. Arrows indicate infiltration and dotted area points to necrosis. **e** Serum ALT levels of 8-week-old NEMO^f/f^, NEMO^Δhepa^, Fas^*lpr*^, and NEMO^Δhepa^/Fas^*lpr*^ mice were determined. Results are expressed as mean ± SEM (*n* = 50 mice, *p* < 0.001–0.01)

Macroscopic appearance of NEMO^Δhepa^/Fas^*lpr*^ livers was normal. Eight-week-old NEMO^Δhepa^ livers are histologically characterized by lack of lobular disorganization, hepatocellular hyperplasia, hypertrophy, and severe diffuse hepatocellular anisokaryosis with marked increase in the apoptotic and mitotic rate. In turn, no neoplasia was present in the hepatic parenchyma of 8-week-old NEMO^Δhepa^/Fas^*lpr*^ livers markedly characterized by multifocal necrosis (Fig. 1a-d). A significant decrease in markers of liver injury at 8 weeks of age was observed in serum ALT (Fig. 1e) compared with NEMO^Δhepa^ mice. No differences were found in AP and GLDH compared with NEMO^Δhepa^ mice (Supplementary Figure 2a, b).

### Impact of FasL/Fas disruption on cell death and compensatory proliferation in NEMO^Δhepa^ livers

**Fig. 2:**
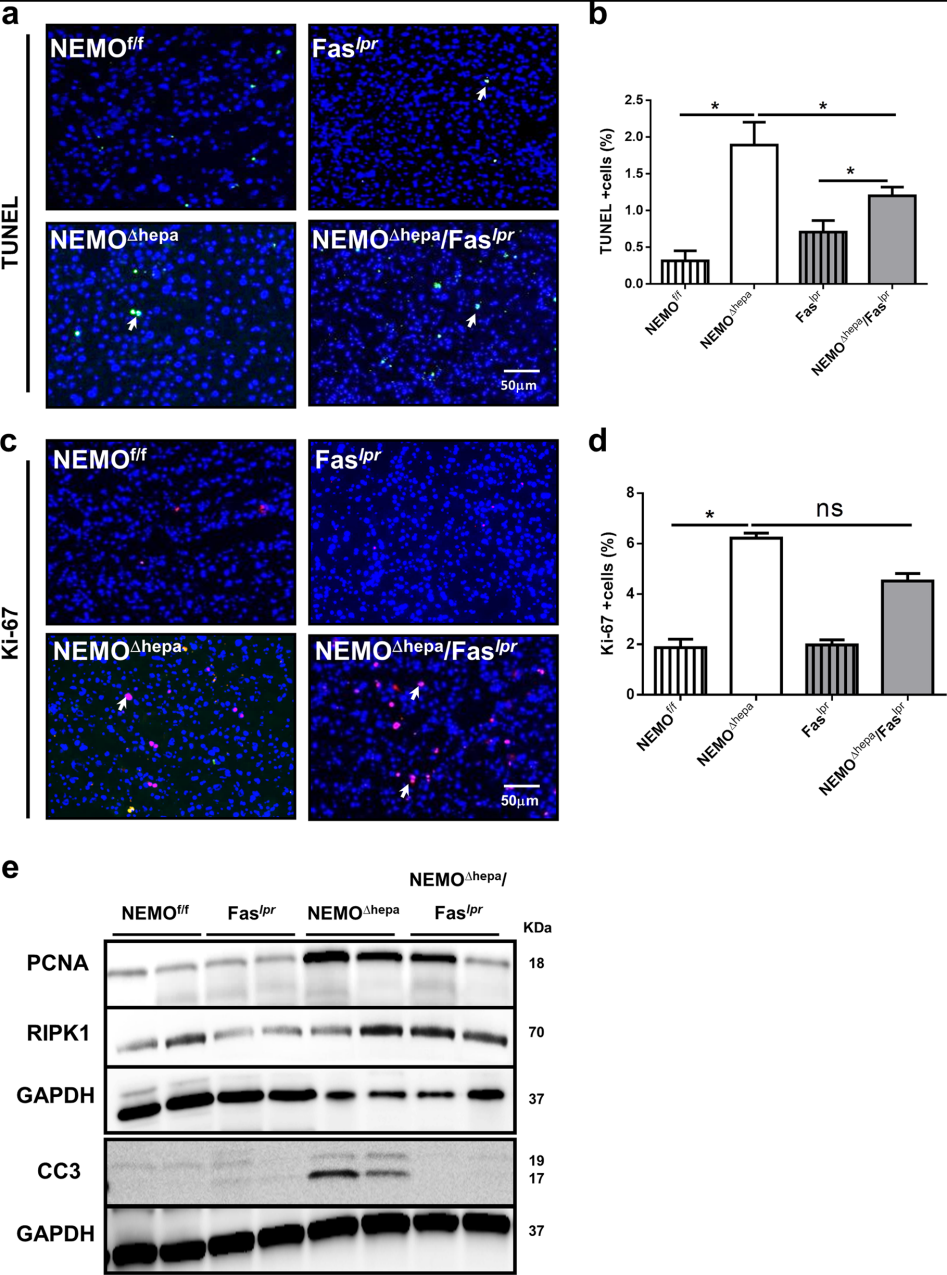
Loss of the FasL/Fas signaling exacerbates cell death and compensatory proliferation in NEMO^Δhepa^ mice. **a** Representative TUNEL staining of 8-week-old NEMO^f/f^, NEMO^Δhepa^, Fas^*lpr*^, and NEMO^Δhepa^/Fas^*lpr*^ livers. **b** TUNEL-positive cells were quantified and graphed. **c** Proliferation was determination by Ki-67-positive cells immunofluorescence. **d** Ki-67-positive cells were quantified and graphed. **e** Expression of PCNA, cleaved Caspase-3 (CC3), and RIPK1 was analysed by immunoblotting of whole liver extracts. GAPDH served as a loading control. Results are expressed as mean ± SEM (*n* = 8 livers, *p* < 0.001–0.05)

The decrease in transaminase levels observed in mutant NEMO^Δhepa^/Fas^*lpr*^ prompted us to investigate the impact of Fas deletion on cell death in NEMO^Δhepa^ mice. Significant differences were evident in TUNEL-positive cells between NEMO^Δhepa^ and NEMO^Δhepa^/Fas^*lpr*^ livers (Fig. 2a, b). Interestingly, the mRNA transcripts of *Bcl2*, a pro-survival protein, was significantly upregulated in NEMO^Δhepa^/Fas^*lpr*^ compared with NEMO^Δhepa^ livers (Supplementary Figure 2c). Since enhanced cell death triggers compensatory proliferation in NEMO^Δhepa^ livers^8^, we further tested whether disruption of FasL/Fas signalling would have an impact on cell proliferation in NEMO^Δhepa^/Fas^*lpr*^ mice. By using cell cycle markers for total cell cycle activity (Ki-67), we found a clear trend towards reduced cell proliferation and significantly lower cell cycle activity (CcnD1) in NEMO^Δhepa^/Fas^*lpr*^ livers compared with NEMO^Δhepa^ (Fig. 2c, d; Supplementary Figure 2d). Interestingly, most Ki-67-positive cells were hepatocytes in NEMO^Δhepa^ livers, whereas immune cells were proliferating in the hepatic parenchyma of NEMO^Δhepa^/Fas^*lpr*^ animals (Fig. 2c, d). Additionally, PCNA protein levels were augmented in NEMO^Δhepa^ compared with NEMO^Δhepa^/Fas^*lpr*^ livers (Fig. 2e). Moreover, cleavage of Caspase-3—but not protein levels of RIPK1—was absent in NEMO^Δhepa^/Fas^*lpr*^ livers (Fig. 2e). Altogether, these results suggest that Fas signaling might promote apoptotic cell death in NEMO^Δhepa^-deficient livers.

### Loss of Fas attenuates fibrogenesis in NEMO^Δhepa^ livers

**Fig. 3:**
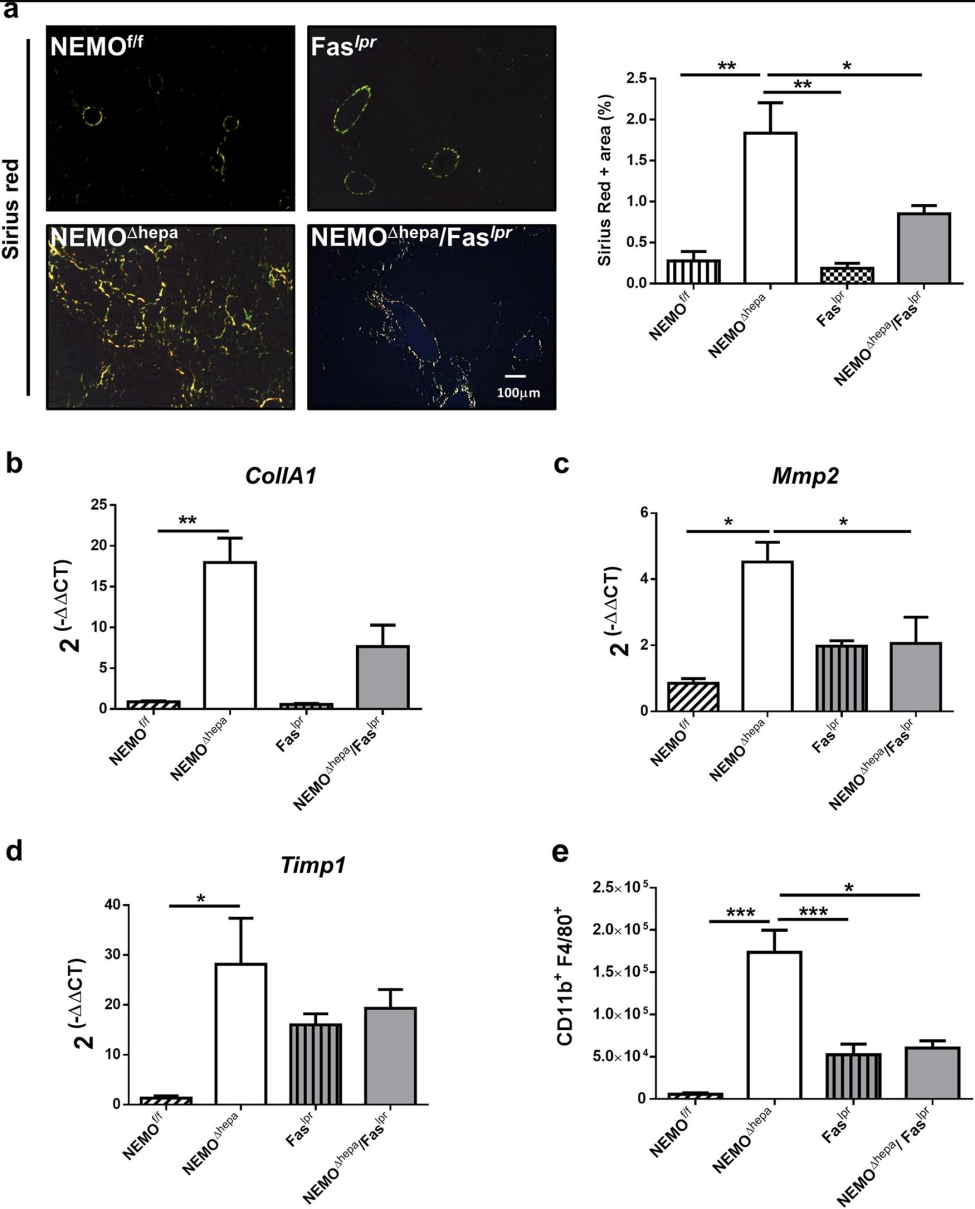
Loss of Fas attenuates liver fibrogenesis in NEMO^Δhepa^ livers. **a** Sirius red staining of paraffin-embedded liver tissue derived from 8-week-old livers of NEMO^f/f^, NEMO^Δhepa^, Fas^*lpr*^, and NEMO^Δhepa^/Fas^*lpr*^ mice. Representative photomicrographs taken under polarized light are shown. SR-positive area was quantifed using ImageJ^©^ and represented. MRNA relative expression of **b** Collagen-1A1, **c** Mmp2, and **d** Timp1 was quantified by RT-PCR. **e** Percentage of F4/80^hi^CD11^hi^-positive cells of each mouse strain was assessed using FACS analysis. Results are expressed as mean ± SEM (*n* *=* *8*, *p* < 0.001–0.01)

Fas might attenuate the effect of gut-derived products on liver injury. Therefore, we next aimed to investigate if this finding might also affect liver disease progression and evaluated its relevance for liver fibrogenesis in 8-week-old animals. Interestingly, deletion of Fas attenuated liver fibrosis in NEMO^Δhepa^. We first measured hepatic fibre formation by Sirius red staining and quantification (Fig. 3a, b), and determined additional well-characterized pro-fibrotic markers, such as Collagen IA1, MMP2, and TIMP1 mRNA expression (Fig. 3c, d).

The infiltration of distinct immune cell populations directed by chemotactic cytokines is a central pathogenic feature following chronic liver injury^13^. Thus, we characterized the hepatic inflammatory cell populations using qRT-PCR and FACS analysis. CD11b^+^ F4/80^+^-proinflammatory macrophages were significantly decreased in NEMO^Δhepa^/Fas^*lpr*^ mice compared to NEMO^Δhepa^ livers (Fig. 3e). Altogether, these findings suggest that Fas deletion—very likely by changing the sensitivity versus LPS—has an anti-inflammatory effect on NEMO^Δhepa^-derived hepatitis.

To test whether decreased liver injury after Fas^*lpr*^ deletion in IKKγ/Nemo mice had an impact on pro-inflammatory cytokines, we performed qRT-PCR for TNF, a main driver of NEMO^Δhepa^-induced liver injury. Interestingly, TNF mRNA levels were significantly reduced in NEMO^Δhepa^/Fas^*lpr*^ compared with NEMO^Δhepa^ livers (Supplementary Figure 3a). However, no differences were found in TGFβ levels between mouse strains (Supplementary Figure 3b). Therefore, we next sought to investigate immune cell infiltration in 8-week-old NEMO^Δhepa^/Fas^*lpr*^ livers. In addition to FACS analysis, we observed significantly decreased CD11b and F4/80-positive cells in NEMO^Δhepa^/Fas^*lpr*^ compared with NEMO^Δhepa^ hepatic tissue (Supplementary Figure 4a-e). These results demonstrate that attenuated liver inflammation after Fas deletion in NEMO^Δhepa^ livers reduces fibrogenesis, pro-inflammatory macrophages, and expression of pro-inflammatory cytokines e.g., TNF.

### Fas signaling is involved in hepatocarcinogenesis (HCC) in NEMO^Δhepa^ mice

**Fig. 4:**
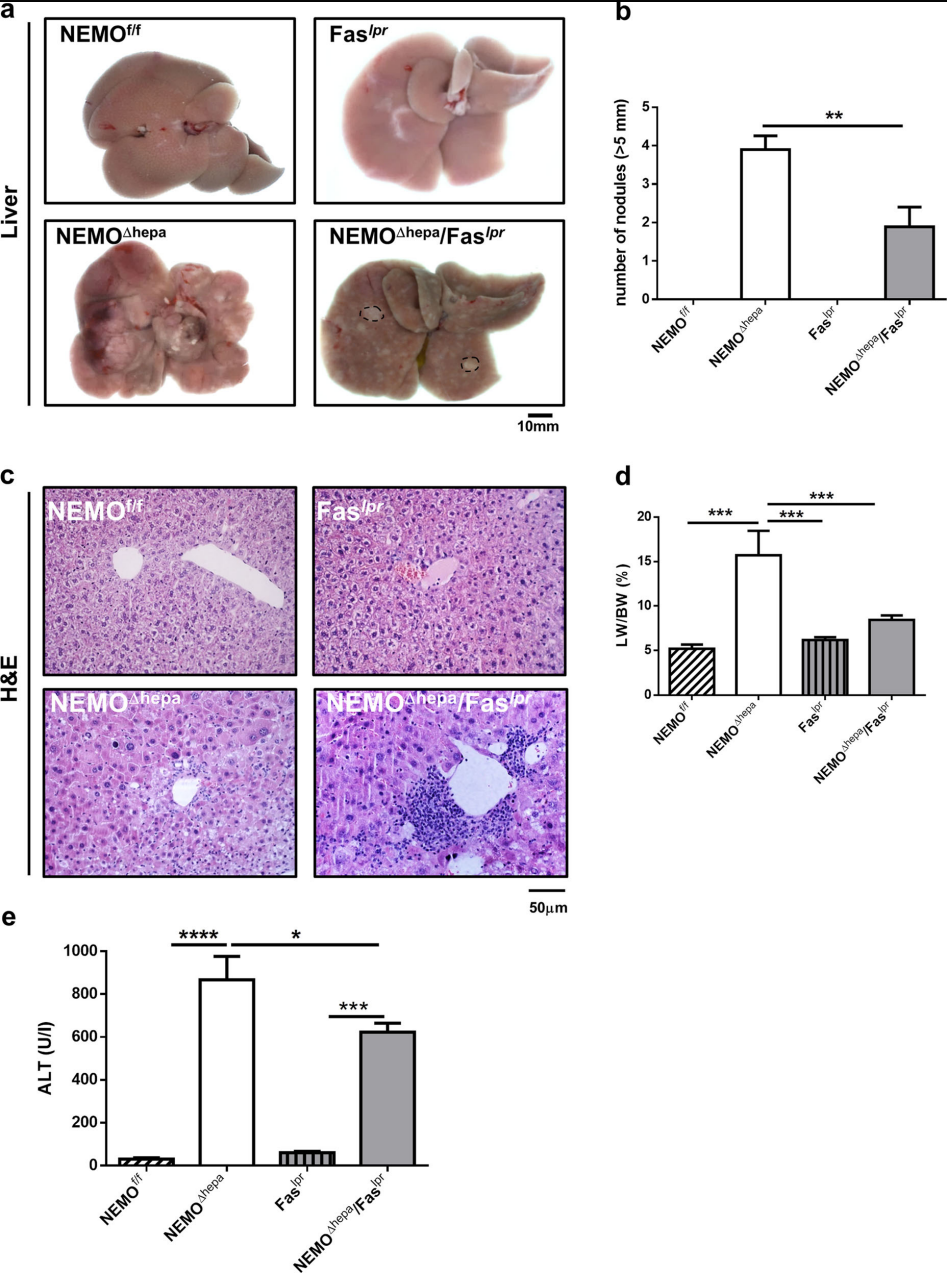
Hepatocarcinogenesis is decreased in 1-year-old NEMO^Δhepa^/Fas^*lpr*^ mice. NEMO^f/f^, NEMO^Δhepa^, Fas^*lpr*^, and NEMO^Δhepa^/Fas^*lpr*^ mice at the age of 12 months were sacrificed and livers extracted and analyzed. **a** Macroscopic appearance of livers. Arrows show visible nodules. **b** The number of nodules (≥5 mm) diameter was quantified. **c** Representative H&E staining of liver sections. **d** Liver weight versus body weight (LW/BW) ratio is shown. **e** Serum ALT was determined in the same mice. Results are expressed as mean ± SEM (*n* *=* *50*, *p < 0.05; **p < 0.01)

Fifty-two-week-old NEMO^Δhepa^ mice develop not only liver fibrosis, but also HCC^8^. Next, we tested the relevance of Fas signaling for liver carcinogenesis. Macroscopically, livers from NEMO^Δhepa^ mice revealed both regenerative nodules and well-defined, large vascularised tumors resulting in a significantly higher liver weight (LW) and liver weight/body weight ratio (LW/BW) compared to NEMO^Δhepa^/Fas^*lpr*^ (Fig. 4).

H&E stainings of 1-year-old NEMO^Δhepa^ mice showed well-differentiated trabecular and solid HCC with nodules of proliferative hepatocytes with disturbed liver architecture and hepatocellular lipid storage, whereas NEMO^Δhepa^/Fas^*lpr*^ showed no neoplasia but atypia and hepatic hyperplasia accompanied by strong perivascular infiltration. Of note, lymphocyte accumulation was also found in Fas^*lpr*^ livers (Table 1). Concomitantly, NEMO^Δhepa^/Fas^*lpr*^ exhibited decreased number of nodules and significantly lower LW/BW ratio compared with NEMO^Δhepa^ livers (Fig. 4a–d).

**Table 1:** Histopathological characteristics of the different mouse groups

	Nemo	Nemo/Fas^*lpr*^	Nemo/Fas^*lpr*^/TNFR1^−/−^
Neoplasia	HCC^a^^1^	No^a^^2^	No^a^^3^
Anisokaryosis	4.17 ± 0.98	4.38 ± 0.51	2.88 ± 1.25^c, d^
Altered foci	2.17 ± 1.17	2.00 ± 1.07	0.62 + 0.91^d^
Mitosis/HPF (40 × )	1.75 ± 2.47	1.43 ± 0.79	0.83 ± 1.33
Cellular hypertrophy	2.5 ± 1.22	2.25 ± 0.46	1.13 ± 0.99^d^
Dysplasia	2.5 ± 1.27	2.30 ± 0.46	1.00 ± 0.93^d^
Oval cell proliferation	2.17 ± 0.98	1.5 ± 0.53	0.87 ± 0.64^c, d^
Lymphoid aggregates/lymphocytic inflammation	0.50 ± 0.00	1.00 ± 0.00	0.86 ± 0.89
Apoptosis	1.50 ± 0.70	1.28 ± 0.48	0.67 ± 0.51
Additional lesions	Mild lipidosis diffuse vaculopathy	Multifocal necrosis	Biliary atresia early lymphomas

^a1^Well-differentiated trabecular or solid HCCs

^a2^Early hepatocellular adenomas

^a3^Single well-differentiated trabecular and glandular HCC

^c^Nemo versus Nemo/Fas^*LPR*^/TNFR1^−/−^

^d^Nemo/Fas^*LPR*^ versus Nemo/Fas^*LPR*^/TNFR1^−/−^

Additionally, a tendency towards reduced ALT was observed in 52-weeks-old NEMO^Δhepa^/Fas^*lpr*^ compared with NEMO^Δhepa^ mice (Fig. 4e). Together, these results indicate that loss of Fas protects against tumorigenesis in the NEMO^Δhepa^ experimental model of chronic liver injury.

### TNFR1 deficiency reduces tumor load in NEMO^Δhepa^/Fas^*lpr*^ livers

Since deletion of TNFR1 is highly beneficial not only to NEMO^Δhepa 10^ but also to NEMO^Δhepa^/p21^−/−^ knockout mice^14^, we generated NEMO^Δhepa^/Fas^*lpr*^/TNFR1^−/−^ triple knockout (TKO) mice and investigated the progression of chronic liver disease in these animals. Eight-week-old TKO mice displayed increased spleen size and a significantly larger number of peritoneal lymph nodes (Supplementary Figure 5a–c). Macroscopically, 8-week-old NEMO^Δhepa^/Fas^*lpr*^/TNFR1^−/−^ livers revealed reduced number of nodules, accompanied by significantly ameliorated ALT, AP, and GLDH compared with NEMO^Δhepa^ and NEMO^Δhepa^/Fas^*lpr*^ mice (Supplementary Figure 6a–d).

**Fig. 5:**
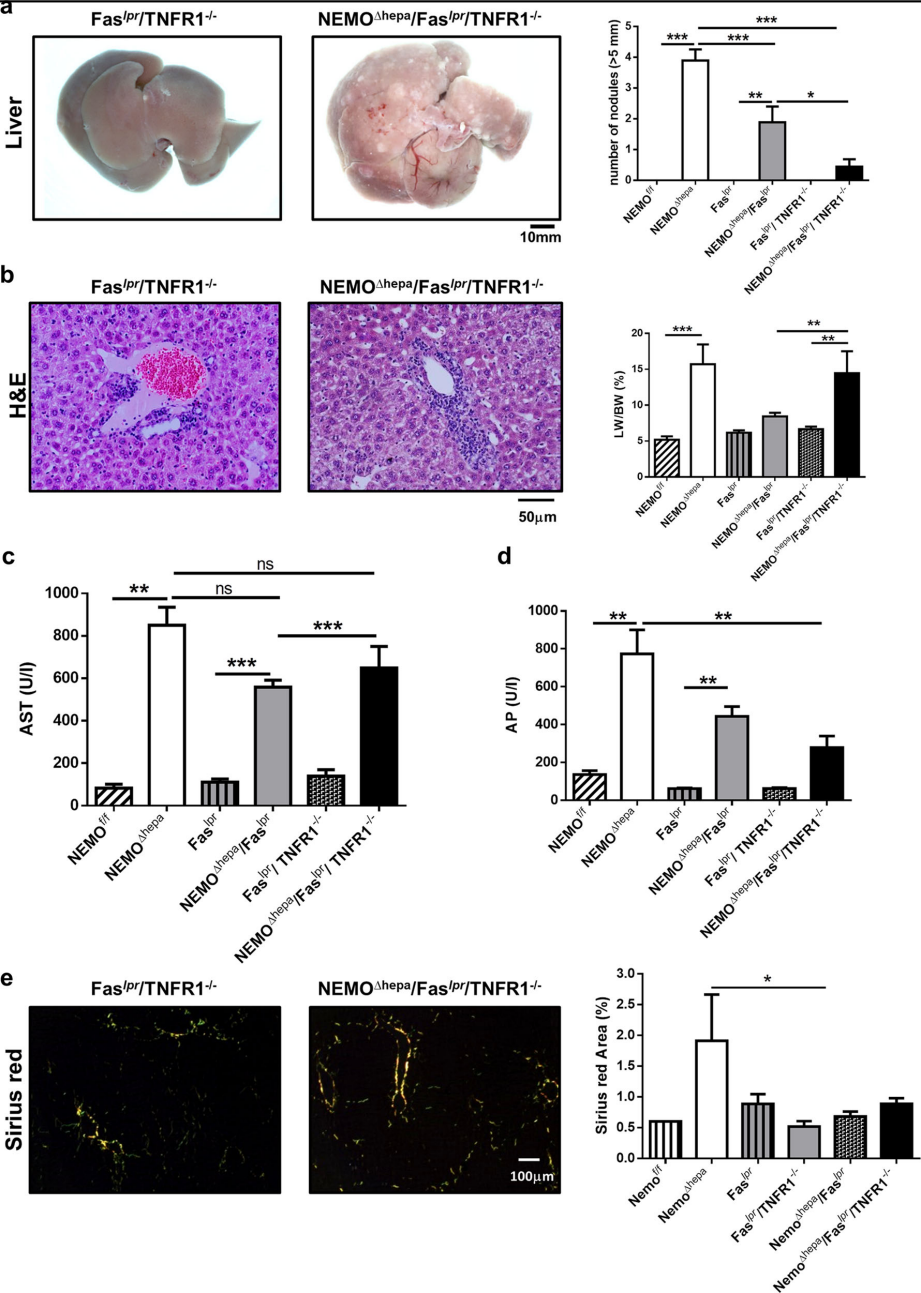
Deletion of TNFR1 amplifies the protective effect of Fas signalling blockade in NEMO mice. Fas^*lpr*^/TNFR1^−/−^ and NEMO^Δhepa^/Fas^*lpr*^/TNFR1^−/−^ mice at the age of 12 months were sacrificed and livers extracted and analyzed. **a** Macroscopic appearance of livers. Arrows show visible nodules. The number of nodules (≥5 mm) diameter was quantified. **b** Representative H&E staining of liver sections. Liver weight versus body weight (LW/BW) ratio is shown. Serum AST (**c**) and AP (**d**) were determined in the same mice and compared to 1-year-old NEMO^f/f^, NEMO^Δhepa^, Fas^*lpr*^, and NEMO^Δhepa^/Fas^*lpr*^. **e** Sirius red staining of paraffin-embedded liver tissue was performed and photomicrographs were taking. SR-positive area was quantifed using ImageJ^©^ and represented including 1-year-old NEMO^f/f^, NEMO^Δhepa^, Fas^*lpr*^, and NEMO^Δhepa^/Fas^*lpr*^. Results are expressed as mean ± SEM (*n* *=* *25*, *p < 0.05; **p < 0.01)

Histopathological examination indicated that 1-year-old NEMO^Δhepa^/Fas^*lpr*^/TNFR1^−/−^ knockout mice presented no signs of neoplasia, early lymphomas, and reduced number of nodules (Table 1). However, no differences in LW/BW ratio (Fig. 5a, b) or important changes in serum AST levels albeit significantly decreased AP levels in 52-week-old NEMO^Δhepa^/Fas^*lpr*^/TNFR1^−/−^ compared with NEMO^Δhepa^, and NEMO^Δhepa^/Fas^*lpr*^ mice were observed (Fig. 5c, d). Interestingly, NEMO^Δhepa^/Fas^*lpr*^/TNFR1^−/−^ knockout mice displayed similar inflammation as NEMO^Δhepa^/Fas^*lpr*^ livers as observed by Sirius red staining and quantification (Fig. 5e, Supplementary Figure 7). Altogether, these data show that TNF deficiency reduces tumorigenesis in NEMO^Δhepa^/Fas^*lpr*^ livers.

**Fig. 6:**
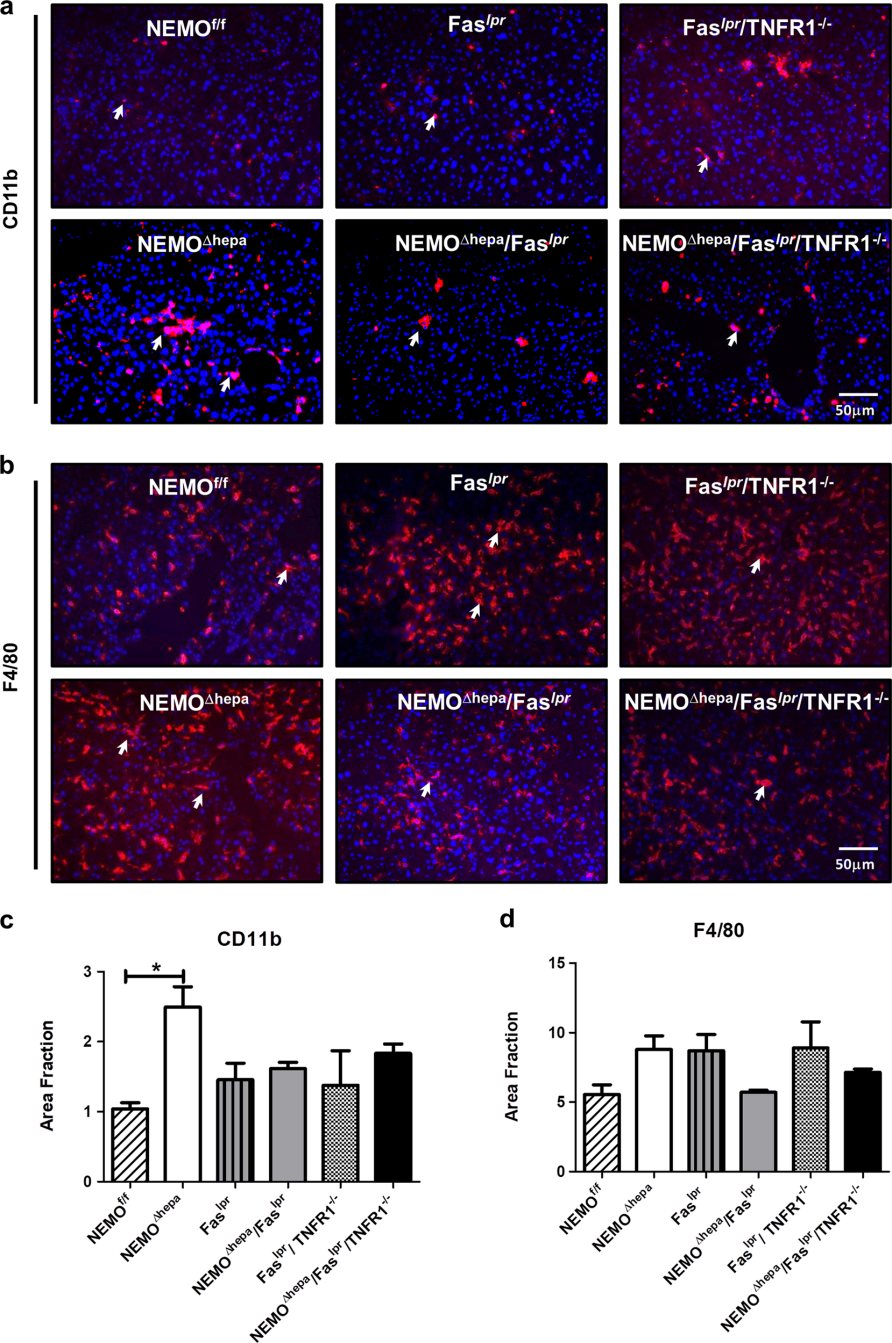
Impact of Fas and TNFR1 deletion on liver infiltrating inflammatory cells in NEMO^Δhepa^ mice. **a** Representative CD11b immunostaining of 52-week-old NEMO^f/f^, NEMO^Δhepa^, Fas^*lpr*^ NEMO^Δhepa^/Fas^*lpr*^, Fas^*lpr*^/TNFR1^−/−^, and NEMO^Δhepa^/Fas^*lpr*^/TNFR1^−/−^ livers. **b** The positive Area Fraction for CD11b was quantified in ImageJ^©^ and graphed. **c** Representative F4/80 immunostaining of 52-week-old NEMO^f/f^, NEMO^Δhepa^, Fas^*lpr*^ NEMO^Δhepa^/Fas^*lpr*^, Fas^*lpr*^/TNFR1^−/−^, and NEMO^Δhepa^/Fas^*lpr*^/TNFR1^−/−^ livers. **d** The positive Area Fraction for F4/80 was quantified in ImageJ^©^ and graphed

In order to better characterize the TNFR1-mediated effect in NEMO^Δhepa^/Fas^*lpr*^, the inflammatory profile of 52-week-old livers was exhaustively investigated. The number of CD11b-positive cells was statistically significantly higher in 52-week-old NEMO^Δhepa^ livers compared to WT animals (Fig. 6c). Elevated F4/80 cells were also found in NEMO^Δhepa^ livers. In contrast, a trend towards reduction of CD11b and F4/80 cells in 52-week-old NEMO^Δhepa^/Fas^*lpr*^ livers was observed (Fig. 6a–d). However, no differences in cell infiltration were evident between NEMO^Δhepa^/Fas^*lpr*^ and NEMO^Δhepa^/Fas^*lpr*^/TNFR1^−/−^ animals, suggesting that TNFR1 might be more involved in modulating tumorigenesis.

## Discussion

Apart from TNFR1 and TRAILR/D5, FasL/Fas is the third death receptor of the TNF receptor superfamily that can activate caspase-8-mediated apoptosis^15^. Previously, we first showed that loss of TRAILR does not improve experimental hepatitis as others have recently confirmed^10,16^. Next, we observed that TNFR1 deletion is beneficial for the progression of chronic liver injury in NEMO^∆hepa^ mice^10,14^. Moreover, we specifically showed that TNFR1 deletion in hepatocytes is protective, whereas TNFR1 inactivation in bone-marrow-derived cells might be deleterious in this experimental model of chronic liver disease. In contrast, a recent study suggested no role for TNFR1 in hepatocytes in the same model albeit showing a clear trend towards reduced transaminases and tumorigenesis^16^.

Since its first description in 1989, the FasR and FasL system has become the best-characterized extracellular system triggering apoptosis^17^. Accumulating evidence highlighted the importance of FasL/Fas expression in the pathogenesis of many gastrointestinal diseases including Wilson’s disease, cholestatic liver disease, alcoholic hepatitis, non-alcoholic steatohepatitis (NASH), and hepatocellular carcinoma (HCC)^18,19^. In the present study, we hypothesized that Fas mediates TNF-induced cell death in NEMO-deficient hepatocytes thus triggering the progression of chronic liver disease and end-stage HCC.

As previously described^20^, Fas mice (Fas^*lpr*^), mutant for Fas, develop splenomegaly, lymphadenopathy, and glomerulonephritis, including mononuclear cell infiltration, and display accumulation of CD4^−^CD8^−^ T lymphocytes in peripheral organs such as the liver. These mice develop autoimmunity and are an excellent model of systemic lupus erythematosus. Interestingly, NEMO^Δhepa^/Fas^*lpr*^ displayed larger spleens than Fas^*lpr*^ mice but no differences in number of lymph nodes. On the other hand, Fas^*lpr*^/TNFR1^−/−^ and NEMO^Δhepa^/Fas^*lpr*^/TNFR1^−/−^ animals exhibited a larger number of peritoneal nodes.

Defective Fas signalling in NEMO^Δhepa^ livers resulted in decreased serum transaminases and multifocal necrosis. In contrast, NEMO^Δhepa^ with normal Fas signalling displayed high mitotic index, oval cell proliferation, mild lipidosis, and diffuse vasculopathy. FasL and Fas are expressed in NEMO-deficient livers, suggesting that they might be involved in mediating apoptosis in NEMO^Δhepa^ hepatocytes^16^. Thus, we first explored the role of Fas in cell death of NEMO^Δhepa^ mice. Decreased TUNEL-positive cells and absence of Caspase-3 activation were associated with lower compensatory proliferation in NEMO^Δhepa^/Fas^*lpr*^ compared with NEMO^Δhepa^ livers. Moreover, Fas^*lpr*^ do not develop liver hyperplasia, but small amount of Fas protein may still be produced by the *lpr* mutant Fas allele and mice engineered to completely lack Fas protein did exhibit liver hyperplasia^21^. Altogether, neither systemic nor specific ablation of Fas in hepatocytes completely prevent cell death but increased hepatocyte survival in NEMO^Δhepa^ animals.

Next, we measured TNF levels since this proinflammatory cytokine is responsible for cell death in NEMO^Δhepa^ liver^8^. Interestingly, TNF levels were significantly downregulated in NEMO^Δhepa^/Fas^*lpr*^. Moreover, NEMO^Δhepa^/Fas^*lpr*^ livers exhibited decreased liver fibrosis and significantly reduced presence of CD11b^+^ F4/80^+^ cells. Since inflammation is a critical factor for progression of liver injury, these observations suggest that an attenuated inflammatory response, and not reduced Fas-induced apoptosis was the cause for the protective effect in NEMO^Δhepa^ mice^22^.

Previously, our group showed that NEMO^Δhepa^ mice are resistant to Jo2 stimulation, a model of Fas-induced damage^9^. However, direct binding of Jo2 to hepatocytes in vivo is not completely clear^23^, and the effect of Jo2 is not restricted only to the liver; it could also affect other tissues of which cells often express a higher level of Fas than hepatocytes. Additionally, Ehlken^16^, using specific deletion of Fas in liver parenchymal cells, found that Fas is not required for the development of chronic liver damage in NEMO^Δhepa^ mice.

In the present study, we employed NEMO^Δhepa^/Fas^*lpr*^ mice, which carry a mutation in their Fas gene caused by insertion of the *Etn* retrotransposon into intron 2 of this gene^24^. Hence, these result in the expression of a completely defective Fas antigen. Thus, our experiments overcome the underestimated effect of Fas-mediated signaling in other tissues that can directly affect the development of chronic liver disease. Concomitant to our results, Hatano et al.^25^ also showed that NF-κB inhibition sensitizes hepatocytes to Fas-mediated apoptosis. Altogether, our study takes into account overall Fas signaling rather than hepatocyte-specific Fas-mediated apoptosis to explain the phenotype of NEMO^Δhepa^ mice.

The FasL/Fas system is a major mechanism of certain types of cancer cells for avoiding detection and destruction by the immune system through FasL expression^7^. Interestingly, NEMO^Δhepa^/Fas^*lpr*^ displayed reduced tumorigenesis compared to NEMO^Δhepa^ mice. Moreover, in the chronic phase reduced inflammation-driven carcinogenesis and lack of functional T cells play an essential role in leading to reduced disease progression in NEMO^Δhepa^/Fas^*lpr*^ livers, which is associated with reduced fibrosis and liver tumorigenesis.

Besides a direct cytotoxic effect on hepatocytes, the TNF/TNFR1 system might be also required for Fas-mediated cell death, as demonstrated by the increased resistance of TNFR1/2 double knockout mice to Fas-induced fulminant liver injury^26^. Thus, we further assessed the role of TNFR signaling in Fas signaling. As previously described as beneficial in ethanol-mediated liver injury^27^, deletion of TNFR1 in NEMO^Δhepa^/Fas^*lpr*^ animals reduced the tumor load of these mice, indicating that TNFR1 deficiency might modulate tumor load in HCC development.

Therefore, therapeutic approaches aiming to inhibit Fas-mediated liver injury might be relevant in inhibiting inflammation-driven hepatic disease progression including growth of HCC.

## Supplementary information

Supp. Material
