# Supplementary material for: Disruption of the FasL/Fas axis protects against inflammation-derived tumorigenesis in chronic liver disease
Source: Cell Death Dis. 2019 Feb 8;10(2):115. doi: 10.1038/s41419-019-1391-x (PMC6368573; doi:10.1038/s41419-019-1391-x)
Supplement: Supplementary file 1 — Supp. Material [file 41419_2019_1391_MOESM1_ESM.pdf]

SUPPLEMENTARY MATERIAL

SUPPLEMENTARY FIGURE LEGENDS

Supplementary Figure 1. Generation and characterization of NEMO$^{\Delta hepa}$/Fas^{lpr} mice. (**a**) PCR blot of IKKγ/NEMO and Fas shows WT and knockout bands in NEMO$^{f/f}$, NEMO$^{\Delta hepa}$, Fas^{lpr} and NEMO$^{\Delta hepa}$/Fas^{lpr}. (**b**) Representative photograph of spleens and peritoneal lymph nodes of Fas^{lpr} and NEMO$^{\Delta hepa}$/Fas^{lpr} mice. (**c**) The spleen size and (**d**) the number of peritoneal lymph nodes of 8 week-old NEMO$^{f/f}$, NEMO$^{\Delta hepa}$, Fas^{lpr} and NEMO$^{\Delta hepa}$/Fas^{lpr} is represented. Results are expressed as mean ± SEM (*n=25*, **p<0.01; **p<0.01).

Supplementary Figure 2. Markers of liver injury after Fas^{lpr} deletion. (**a**) Serum AP and (**b**) GLDH levels of 8 week-old NEMO$^{f/f}$, NEMO$^{\Delta hepa}$, Fas^{lpr} and NEMO$^{\Delta hepa}$/Fas^{lpr} mice were determined. Results are expressed as mean ± SEM (*n=25*, **p<0.01; **p<0.01).

Supplementary Figure 3. Cell death, proliferation and inflammation markers after deficient FasL/FasR signaling. MRNA relative expression of (**a**) TNF and (**b**) TGFβ was quantified by RT-PCR and graphed.

Supplementary Figure 4. Infiltration of inflammatory cells into the liver. (**a**) FACS blot of proinflammatory macrophages CD11b/F4/80 is shown. (**b**) Representative CD11b staining of 8 week-old NEMO$^{f/f}$, NEMO$^{\Delta hepa}$, Fas^{lpr} and NEMO$^{\Delta hepa}$/Fas^{lpr} livers. (**c**) The positive Area Fraction was quantified in Image J©

and graphed. (**d**) Representative F4/80 staining of the same livers. (**e**) The positive Area Fraction was quantified in Image J© and graphed.

Supplementary Figure 5. Characterization of NEMO$^{\Delta hepa}$/Fas^{lpr}/TNFR1$^{-/-}$ mice. (**a**) Representative photograph of spleens and peritoneal lymph nodes of 8 week-old Fas^{lpr}/TNFR1$^{-/-}$ and NEMO$^{\Delta hepa}$/Fas^{lpr}/TNFR1$^{-/-}$ mice. (**b**) The spleen size and (**c**) the number of peritoneal lymph nodes of 8 week-old Fas^{lpr}/TNFR1$^{-/-}$ and NEMO$^{\Delta hepa}$/Fas^{lpr}/TNFR1$^{-/-}$ compared with NEMO$^{f/f}$, NEMO$^{\Delta hepa}$, Fas^{lpr} and NEMO$^{\Delta hepa}$/Fas^{lpr} is represented. Results are expressed as mean ± SEM (*n=25*, **p<0.01; ***p<0.001).

Supplementary Figure 6. Deletion of TNFR1 further protects against the NEMO-dependent hepatitis. Fas^{lpr}/TNFR1$^{-/-}$ and NEMO$^{\Delta hepa}$/Fas^{lpr}/TNFR1$^{-/-}$ mice at the age of 8 weeks were sacrificed and livers extracted and analyzed. (**a**) Macroscopic appearance of livers. (**b**) Representative H&E staining of liver sections. (**c**) Serum ALT, (**d**) AP and (**e**) GLDH were determined in the same mice and compared to 8 week-old NEMO$^{f/f}$, NEMO$^{\Delta hepa}$, Fas^{lpr} and NEMO$^{\Delta hepa}$/Fas^{lpr} mice. Results are expressed as mean ± SEM (*n=25*, **p<0.05; ***p<0.001).

Supplementary Figure 7. Liver fibrogenesis is further decreased in 1 year-old NEMO$^{\Delta hepa}$/Fas^{lpr}/TNFR1$^{-/-}$ livers. Sirius red staining of paraffin-embedded liver tissue was performed and photomicrographs in polarized and normal light were taken in NEMO$^{f/f}$, NEMO$^{\Delta hepa}$, Fas^{lpr} and NEMO$^{\Delta hepa}$/Fas^{lpr}, Fas^{lpr}/TNFR1$^{-/-}$ and NEMO$^{\Delta hepa}$/Fas^{lpr}/TNFR1$^{-/-}$ mice at the age of 1 year.

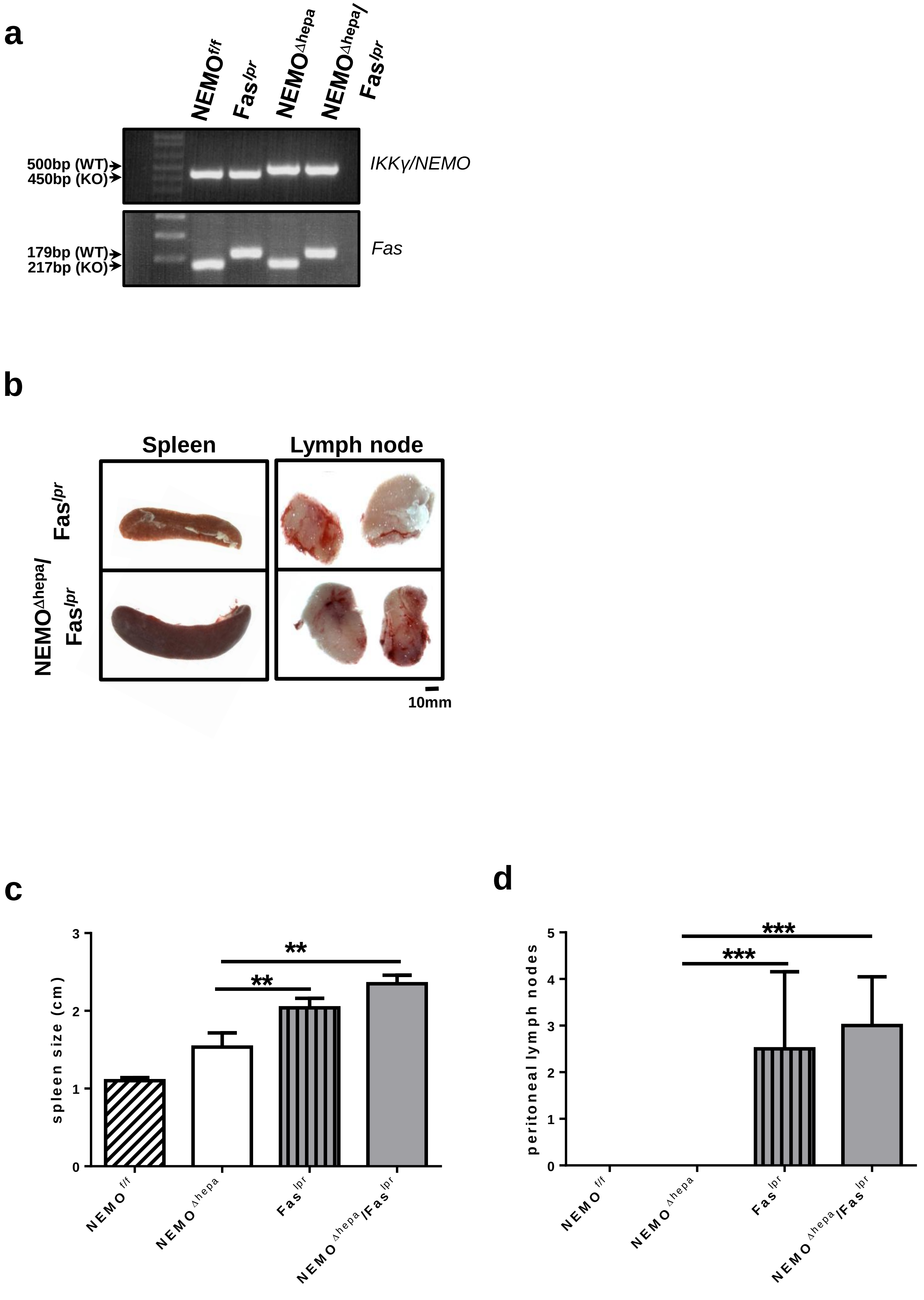

Supplementary Figure 1

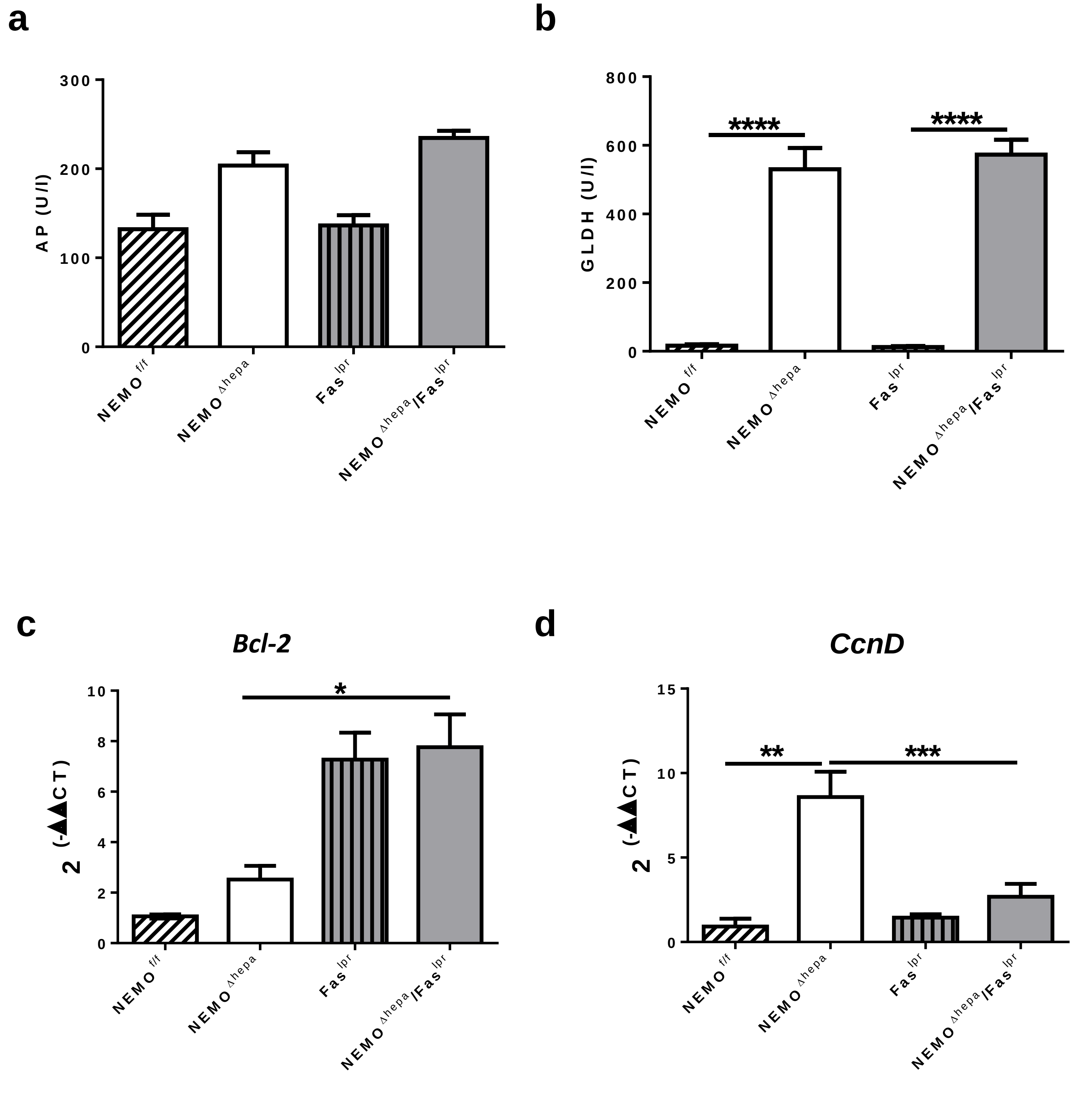

Supplementary Figure 2

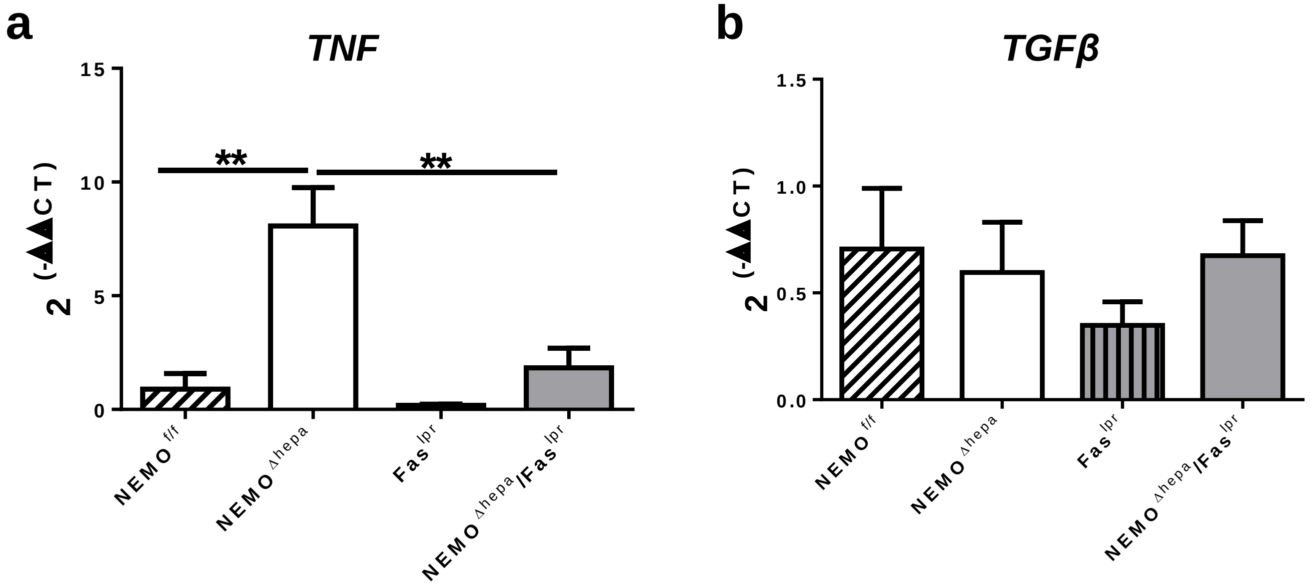

Supplementary Figure 3

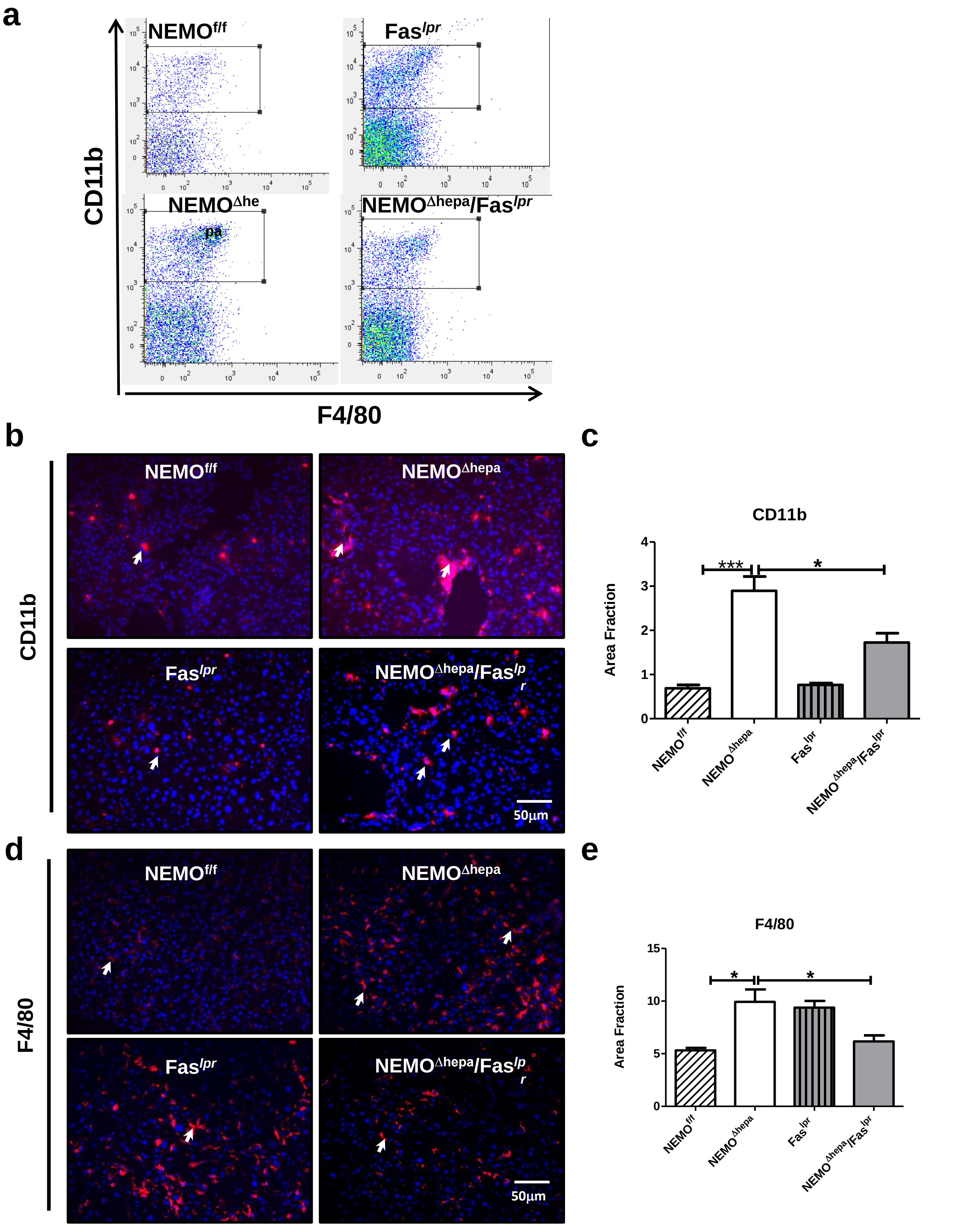

Supplementary Figure 4

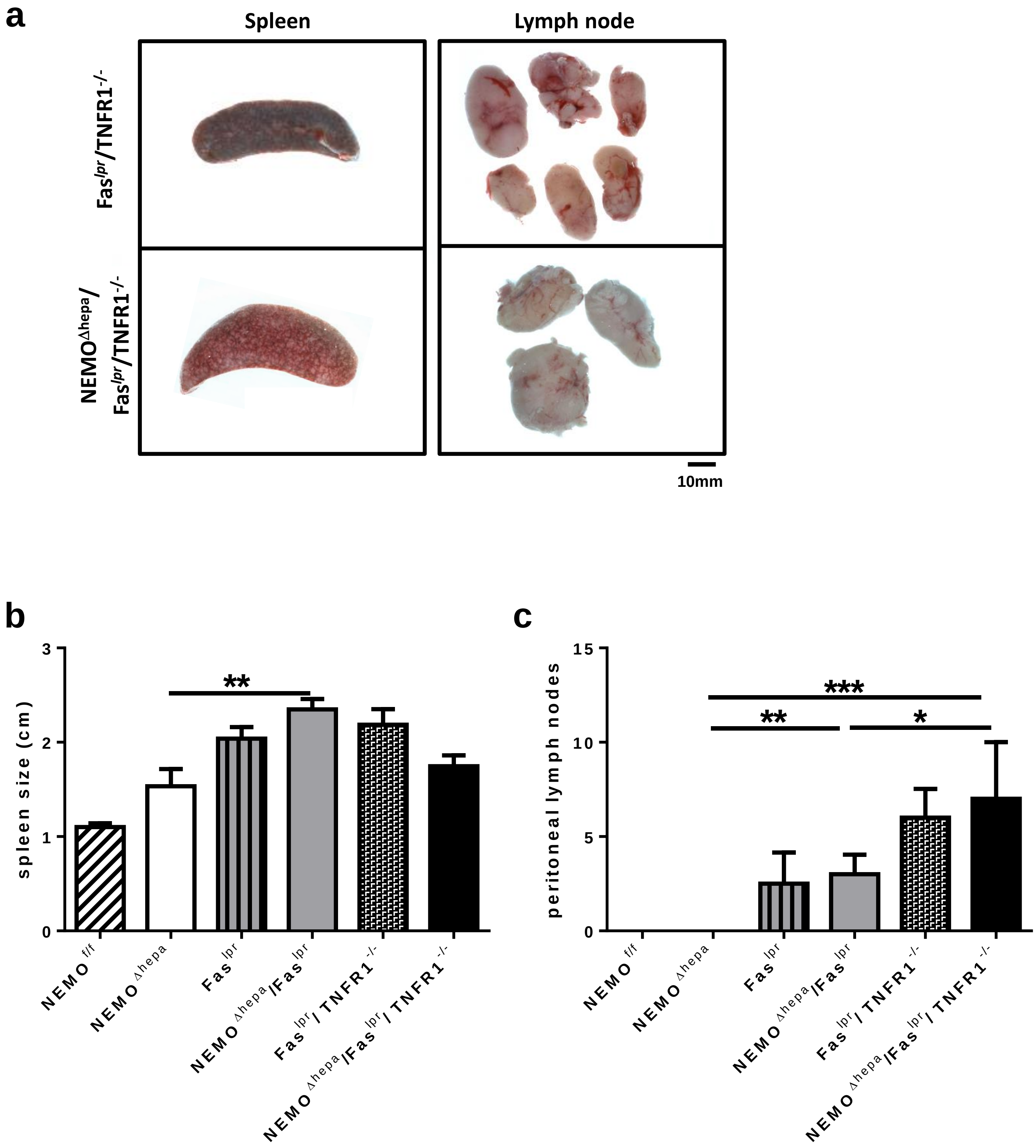

Supplementary Figure 5

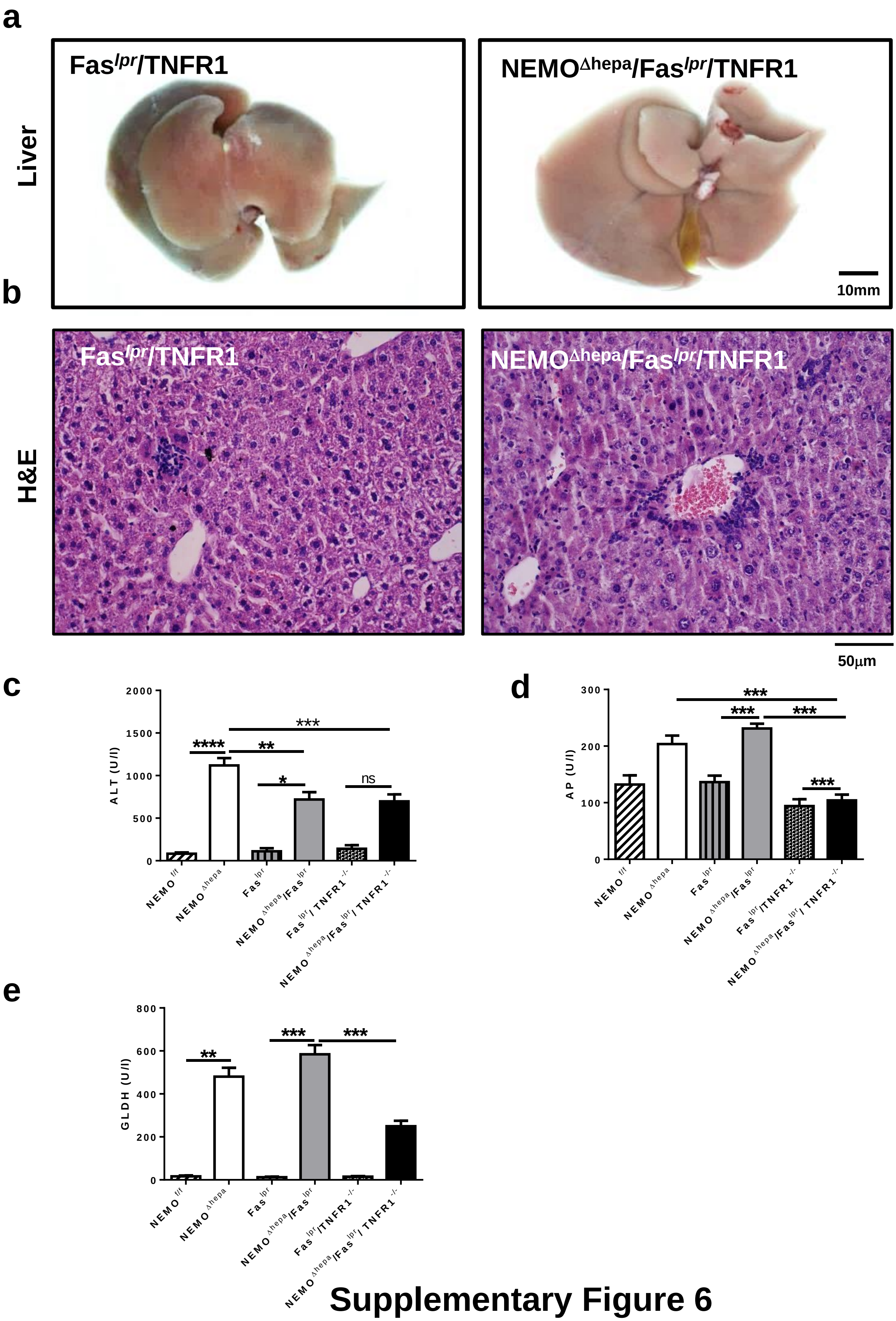

Supplementary Figure 6

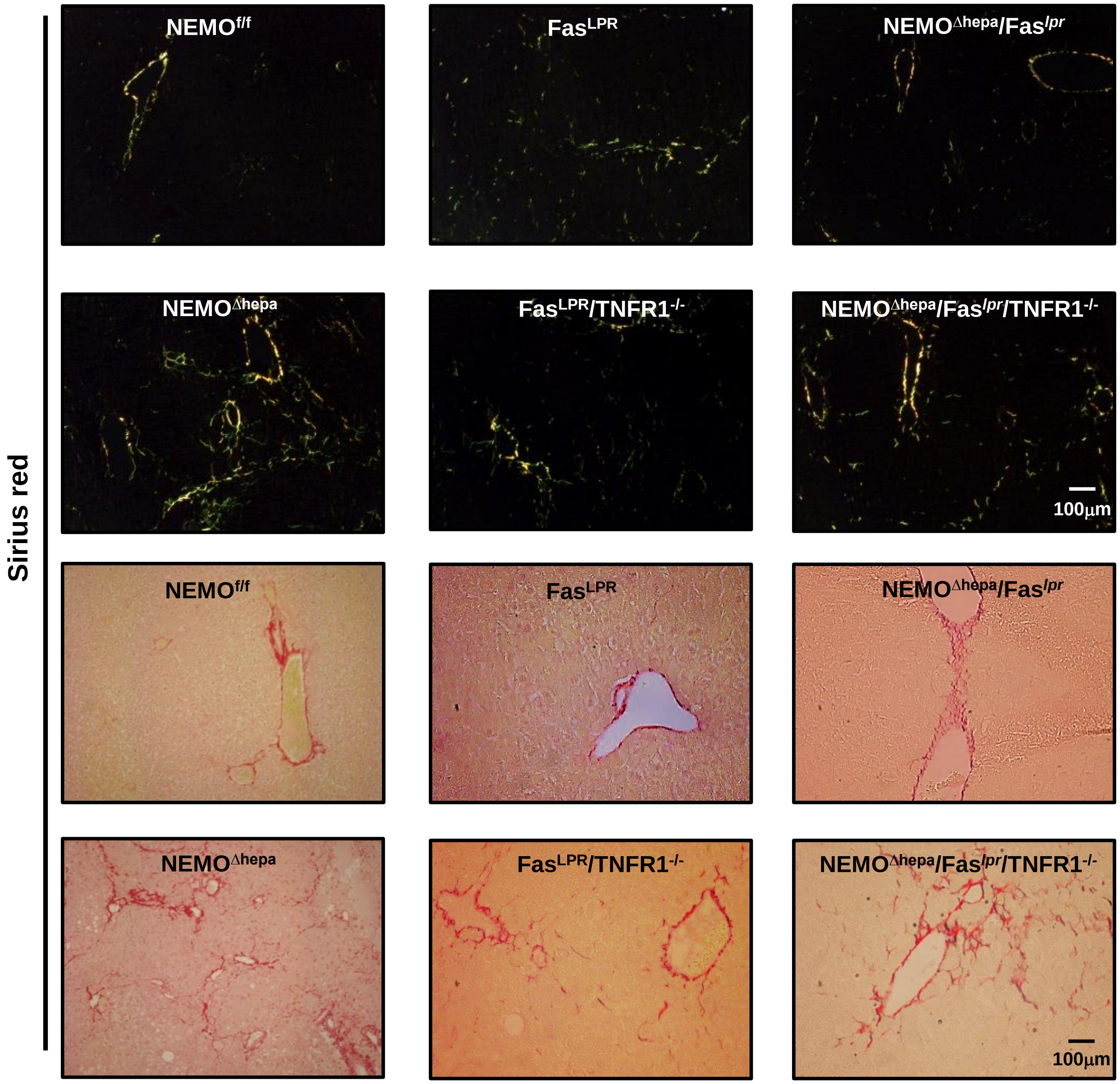

Supplementary Figure 7
